# Kin-recognition and predation shape collective behaviors in the cannibalistic nematode *Pristionchus pacificus*

**DOI:** 10.1371/journal.pgen.1011056

**Published:** 2023-12-14

**Authors:** Fumie Hiramatsu, James W. Lightfoot

**Affiliations:** 1 Max Planck Research Group Genetics of Behavior, Max Planck Institute for Neurobiology of Behavior–caesar, Bonn, Germany; 2 International Max Planck Research School for Brain and Behavior, Bonn, Germany; University of California San Francisco, UNITED STATES

## Abstract

Kin-recognition is observed across diverse species forming an important behavioral adaptation influencing organismal interactions. In many species, the molecular mechanisms involved are difficult to characterize, but in the nematode *Pristionchus pacificus* molecular components regulating its kin-recognition system have been identified. These determine its predatory behaviors towards other con-specifics which prevents the killing and cannibalization of kin. Importantly, their impact on other interactions including collective behaviors is unknown. Here, we explored a high altitude adapted clade of this species which aggregates abundantly under laboratory conditions, to investigate the influence of the kin-recognition system on their group behaviours. By utilizing pairwise aggregation assays between distinct strains of *P*. *pacificus* with differing degrees of genetic relatedness, we observe aggregation between kin but not distantly related strains. In assays between distantly related strains, the aggregation ratio is frequently reduced. Furthermore, abolishing predation behaviors through CRISPR/Cas9 induced mutations in *Ppa-nhr-40* result in rival strains successfully aggregating together. Finally, as *Caenorhabditis elegans* are found naturally occurring with *P*. *pacificus*, we also explored aggregation events between these species. Here, aggregates were dominated by *P*. *pacificus* with the presence of only a small number of predators proving sufficient to disrupt *C*. *elegans* aggregation dynamics. Thus, aggregating strains of *P*. *pacificus* preferentially group with kin, revealing competition and nepotism as previously unknown components influencing collective behaviors in nematodes.

## Author summary

Many species are able to recognize their relatives from other organisms and this influences how they behave with one another. In some species, this kin-recognition ability results in cooperation while in others it can induce more competitive and aggressive behaviors. Importantly, in many species we do not know the mechanisms which regulate these interactions. In the predatory nematode (round worm) *Pristionchus pacificus*, we have previously observed that they attack and kill other nematode larvae but their kin-recognition abilities prevent them from harming their close relatives. Importantly, in *P*. *pacificus* we have an abundance of genetic and molecular tools and can begin to understand how they are able to carry out this recognition behavior. Here, we have shown that their kin-recognition and predatory abilities also influence other interactions including how these nematodes group together. Many nematode species form aggregates to help them find food or to avoid harmful environmental conditions. In *P*. *pacificus*, we have observed that they preferentially aggregate with their close relatives and avoid grouping with more distantly related strains. This may ensure that they give their relatives an advantage within their ecological niche. Therefore, our work will help us understand more about the significance of kin-recognition behaviors and their evolution.

## Introduction

Kin-recognition is observed across many species and is associated with a diversity of different behaviors. This includes behaviors in single celled organisms such as swarming behaviors in bacteria [1], flocculation in yeast [2], and altruism in the amoeba *Dictyostelium* [3–5]. Additionally, behaviors in more complex organisms are also thought to be reliant on kin-recognition including cooperation in insects [6,7] and lizards [8,9], colony fusions events in tunicates [10,11], cannibalism in several amphibian species [12,13], as well as nest mate preference in rodents [14,15]. In nematodes a kin-recognition system was also recently identified in *Pristionchus pacificus*. This is an omnivorous and cannibalistic nematode species and is capable of attacking and killing the larvae of other nematodes as well as other *P*. *pacificus* con-specifics [16,17]. These predatory behaviors likely provide a supplementary nutrient source as well as a mechanism to remove potential competitors from their environment as both territorial behaviors and surplus killing have been described [18–20]. Furthermore, while *P*. *pacificus* can be a voracious predator of other nematode larvae, it avoids killing its close relatives and progeny through the existence of a hypervariable small peptide mediated kin-recognition system [21,22]. However, previous *P*. *pacificus* kin-recognition studies have focused on the interactions between predators and larvae, and little is known of its impact on larger scale group behaviors. Importantly, these larger scale dynamics are likely to occur frequently due to the boom-and-bust life history strategy employed by *P*. *pacificus* in its natural ecological setting which temporarily results in a large concentration of nematodes around a confined niche [23].

While few studies on larger scale collective dynamics have taken place in *P*. *pacificus*, aggregation behaviors have previously been reported in a high-altitude adapted clade as a mechanism to avoid hyperoxia although it is likely other as yet unstudied factors will also induce aggregation in this species [24–27]. Instead, nematode aggregation behaviors have been intensely studied at the molecular and neuronal level in the model nematode *Caenorhabditis elegans*. These behaviors in *C*. *elegans* are induced in response to various factors including hyperoxia, aversive stimuli, food quantity and population density [28–31]. Accordingly, many wild isolates of *C*. *elegans* show aggregation behaviors in which they group together on a bacterial lawn and even exhibit dynamic swarming over longer time periods [32,33]. In contrast, the *C*. *elegans* laboratory reference strain N2 does not aggregate due to the presence of a gain-of-function mutation in the *npr-1* gene and is considered to be a solitary strain [28,34]. This behavioral difference is caused by a single amino acid change in this neuropeptide receptor with gregarious wild isolates carrying a 215F allele version of *npr-1* while in solitary strains a 215V version is found instead which suppresses this behavior. However, both variants have been shown to confer fitness advantages depending on food availability and dispersal strategy [35,36]. In addition to *npr-1*, aggregation is mediated through various receptors found in sensory neurons which depend on the intraflagellar transport (IFT) machinery, several soluble guanylate cyclases and components of the transforming growth factor beta (TGF-β) family [29,30,37,38]. This includes the TGF-β *daf-7* pathway which acts in parallel to *npr-1*. Furthermore, pheromone signaling through small molecule ascarosides also promotes attraction and aggregation which converge on the same neuronal circuits [39–41]. Intriguingly, while the IFT system is essential for aggregation in *P*. *pacificus*, neither *Ppa-npr-1* nor *Ppa-daf-7* are required for these behaviors [26,27,42]. Therefore, aggregation in *P*. *pacificus* is dependent on a distinct mechanistic process that has evolved independently from those described in *C*. *elegans*.

Here, by exploring the specialized high-altitude adapted and aggregating clade of *P*. *pacificus* we identify kin-recognition as an essential component shaping aggregate formation. We find aggregating strains of *P*. *pacificus* preferentially group with their own kin or close relatives and avoid more divergent strains. Moreover, pairwise interactions between distantly related strains reveal that the aggregation ratio is frequently reduced across both strains. This territoriality also extends to interactions of *P*. *pacificus* with other aggregating species including *C*. *elegans* and reveals kin-recognition and competition as a previously unknown component influencing collective behaviors in nematodes.

## Results

**Fig 1:**
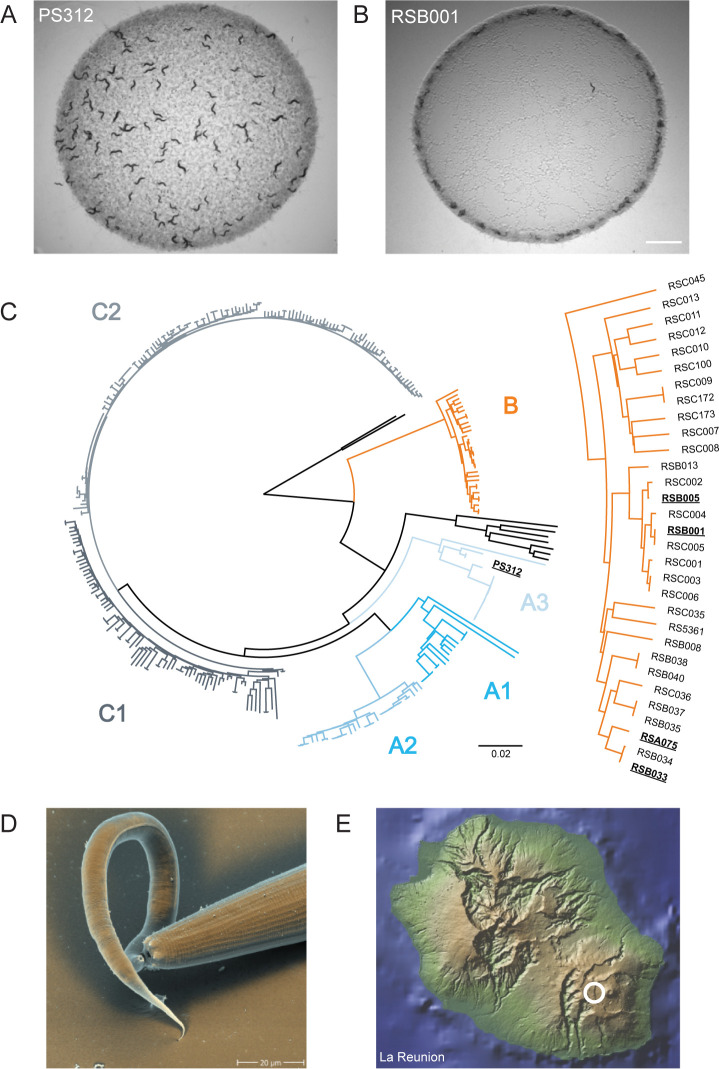
*P*. *pacificus* aggregation behaviors are prevalent in a high altitude adapted clade. (A) The main laboratory *P*. *pacificus* wild type strain, PS312 does not aggregate under standard laboratory conditions. (B) RSB001 is a high altitude adapted strain which aggregates under standard laboratory conditions. Scale bar = 2000 μm. (C) *P*. *pacificus* phylogenetic tree representing the genetic relationship between 323 wild isolates. Image adapted from Rödelsperger et al 2017 [46]. The four strains selected for further analysis are highlighted in bold as well as the main laboratory strain PS312. (D) SEM image of a *P*. *pacificus* predator (large right nematode) killing a *C*. *elegans* larvae (smaller left nematode). (E) Strains selected for analysis were all isolated from the region circled which is a high-altitude environment on La Reunion Island. Figure made with GeoMapApp [44].

Collective behaviors are found in many nematode species indicating its high degree of evolutionary conservation [43]. Conversely, in *P*. *pacificus* most strains behave in a solitary manner with aggregation only predominantly observed in one of the main *P*. *pacificus* evolutionary lineages referred to as clade B [25]. However, it is likely that other unexplored factor may also induce aggregation in this species (Fig 1A–1C). Strains belonging to this clade are frequently found in a high-altitude environment and have adapted to lower oxygen concentrations [25]. As such, under normal laboratory conditions, aggregate formation is induced and animals localize at the border of the bacterial lawn to avoid hyperoxia [24–27]. Furthermore, unlike in *C*. *elegans* this aggregation behavior is observed even in the absence of a bacterial food source with the exception of the *C*. *elegans* dauer state (S1 Fig) [31]. Importantly, like all other strains of *P*. *pacificus* sampled so far, isolates from these locations are also predatory and kill the larvae of other nematodes including other *P*. *pacificus* con-specifics (Fig 1D). They also possess kin-recognition behaviors which prevent the killing of their own progeny and close relatives while enabling the killing of more distantly related strains [21,22]. Therefore, we exploited the *P*. *pacificus* clade B propensity to aggregate to investigate the influence of kin-recognition and its associated predation on group behaviors in these nematodes. Accordingly, we selected four strains from one high altitude location to investigate their interactions and ability to form aggregates together. These strains were all originally isolated from the same area on the Nez de Bœuf volcano peak (>2089 m above sea level) on La Reunion Island (Fig 1E) [44] and as such also represent potentially ecologically relevant interactions. We selected RSB001 as it has previously formed the basis of numerous molecular studies [25,27,45,46], as well as a close relative RSB005. We also selected another pair of strains (RSA075 and RSB033) which are distantly related to RSB001 and RSB005 but are closely related to one another (Fig 1C).

### Kin-recognition and predation influence aggregation behaviors

**Fig 2:**
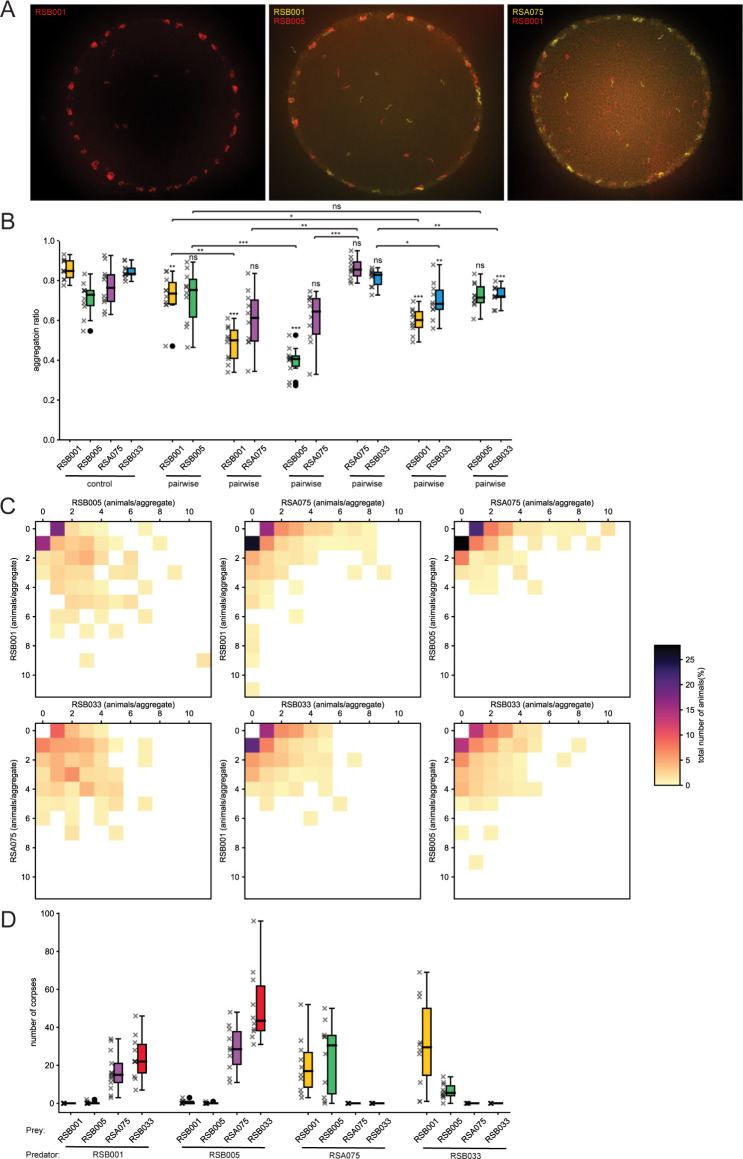
*P*. *pacificus* aggregates with kin and displaces non-kin. (A) Representative images of the aggregation phenotypes of the *P*. *pacificus* strain RSB001 control as well as mixed cultures of RSB001 with RSB005 and RSB001 with RSB075. Scale bar = 2000 μm. (B) Quantification of aggregation ratios of *P*. *pacificus* cultures including RSB001, RSB005, RSA075 and RSB033 controls as well as all pairwise permutations. Aggregation is disrupted between more distantly related strains. Significant decreases in aggregation ratios were observed in assays between distantly-related strains. Statistical tests were performed against control, unless there are bars above the boxplots for any between-pair comparisons (Mann-Whitney-U-test with Bonferroni corrections). n = 10 per condition. (C) Heatmaps of pairwise interactions revealing the proportion of each strain in an aggregate. The two axes each represents the number of animals from the two interacting strains present in an aggregate, with the color indicating the percentage of the population found there. (D) Quantification of predation assays revealing killing between all possible pairwise permutations. Increased killing is observed between more distantly related strains. n = 21 for RSB001 predators on RSB001 prey, n = 15 for RSB001 predators on RSB005 prey, n = 13 for RSB001 predators on RSA075 prey, RSB005 predators on RSB005 prey, and n = 10 for each of the other conditions.

Having identified aggregating strains with differing degrees of genetic relatedness, we next explored if aggregation behaviors occurred between these mixed *P*. *pacificus* populations. To assess this, we established aggregation assays in which 120 worms (60 worms from each strain) were placed onto a defined OP50 *Escherichia coli* bacterial lawn of 70 μl for 3 hrs. In order to differentiate and define strain specific behavioral interactions in mixed populations, we utilized a previously established staining method using fluorescent vital dyes that has been demonstrated to have no effect on the animal health [47] or its aggregation behavior (S2A–S2C Fig). Under these conditions, all four strains aggregated strongly in control assays consisting of only their own strain compared to a solitary strain. (Figs 2A, 2B and S2B–S2E). However, in mixed assays consisting of two strains, we observed striking differences in aggregation depending on the combination of strains assessed. Specifically, aggregates between populations of the relatively closely related pair RSB001 and RSB005 and the other related pair RSA075 and RSB033 were consistent with controls and mostly aggregated together. However, assays between RSB001 or RSB005 together with the more distantly related RSA075 or RSB033, resulted in a significant decrease in aggregate formation (Fig 2A and 2B and S1 Movie). Furthermore, the composition of aggregates also changed in mixed populations with aggregation in one strain frequently reduced more than in the opposing strain (Fig 2B and 2C). Therefore, between strain competition is a key element defining aggregate composition with *P*. *pacificus* preferentially grouping with their own kin and close relatives and not more distantly related strains.

As aggregation behaviors were disrupted between more distantly related strains but not close relatives, we next investigated if this may be due to aggressive predatory behaviors between strains. *P*. *pacificus* attacks other nematodes including other *P*. *pacificus* strains which can result in the killing of these larvae; however, the thicker cuticle of the adults usually prevents fatal interactions and instead induces an escape response (S2 Movie) [16,19]. Additionally, the kin-recognition system prevents any aggressive interactions between a strain’s own progeny as well as their close relatives [21,22]. Therefore, we utilized previously established assays [16] to determine the predation outcome between our selected strains. We observed no killing of self-progeny in all strains as expected and evidence of only very limited predatory interactions between the closely related RSB001 and RSB005 as well as RSA075 together with RSB033. However, predation between any of the more distantly related strains resulted in an aggressive response with large numbers of larvae killed (Fig 2D). Therefore, between strain aggregation is consistent with their kin-recognition and associated predatory interactions. Thus, closely related strains such as RSB001 and RSB005 as well as RSA075 and RSB033 perceive one another as kin, do not attack and are capable of aggregating together. Conversely, more distantly related strains are regarded as non-kin, aggressively predate one another and aggregate formation is disrupted.

### Phenotypic plasticity and predatory interactions enforce kin aggregation

**Fig 3:**
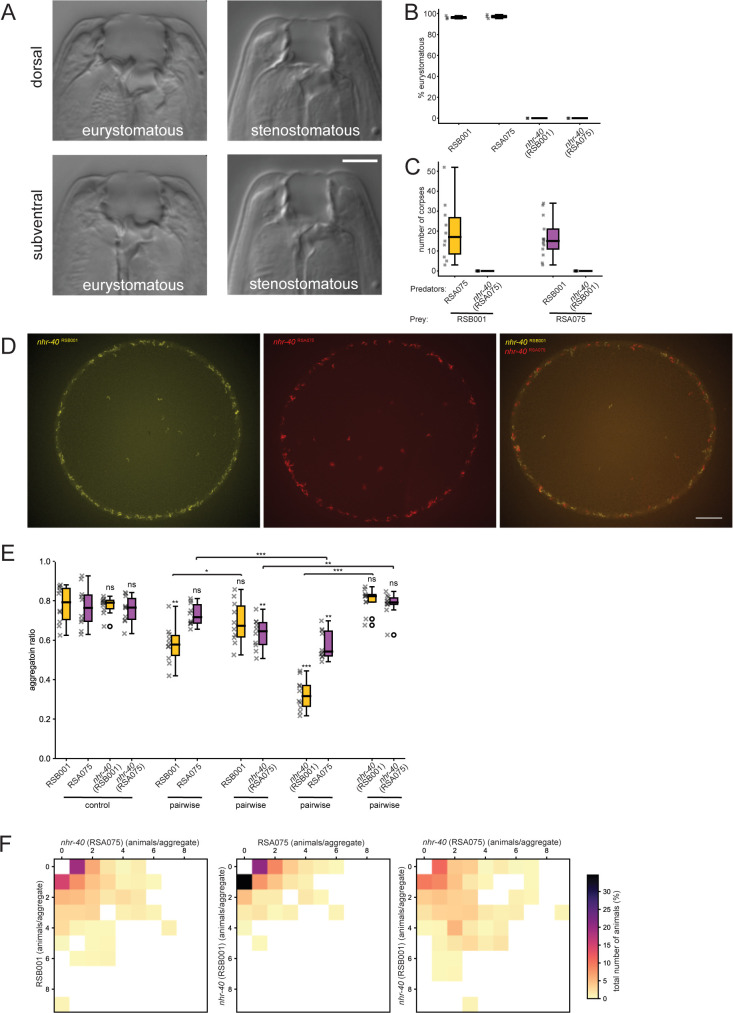
Mouth form plasticity and associated predation behaviors influence aggregation. (A) *P*. *pacificus* mouth dimorphism. The eurystomatous mouth has a wide buccal cavity with two teeth and is omnivorous feeding on both bacteria and other nematodes while the stenostomatous mouth is narrow with a single tooth and microbivorous. Scale bar = 5 μm. (B) Mutations in *Ppa-nhr-40* in both RSB001 and RSA075 change the mouth form frequency from highly Eu to 100% St. (C) Killing assays comparing the ability of RSA075 and *Ppa-nhr-40* ^RSA075^ to predate upon RSB001 prey and the reciprocal assays of RSB001 and *Ppa-nhr-40* ^RSB001^ to predate upon RSA075 prey. Mutations in *Ppa-nhr-40* ^RSB001^ and *Ppa-nhr-40* ^RSA075^ remove the ability of these strains to predate one another. n = 10 per condition. (D) Representative images of the aggregation phenotypes of the *P*. *pacificus Ppa-nhr-40* ^RSB001^ and *Ppa-nhr-40* ^RSA075^ controls as well as mixed cultures of *Ppa-nhr-40* ^RSB001^ and *Ppa-nhr-40* ^RSA075^ which aggregate together. Scale bar = 2000 μm. (E) Quantification of aggregation ratios in *P*. *pacificus* cultures including of RSB001, RSA075, *Ppa-nhr-40* ^RSB001^, and *Ppa-nhr-40* ^RSA075^ controls as well as all pairwise permutations. Significant decreases in aggregation ratios of *Ppa-nhr-40* mutants were observed when assays were performed between the mutants and the wildtype of a distantly-related strain. Statistical tests were performed against control, unless there are bars above the boxplots for any between-pair comparisons (Mann-Whitney-U-test with Bonferroni corrections). n = 10 replicates. (F) Heatmaps of pairwise interactions between different combinations of *Ppa-nhr-40* mutants and the corresponding wildtype strains, revealing the proportion of each strain in an aggregate.

We next investigated the significance of the non-kin associated aggressive interactions on aggregation behaviors in more detail by removing the ability of these strains to predate one another. One mechanism to suppress the predatory behaviors in *P*. *pacificus* is through manipulating their phenotypically plastic mouth as during development *P*. *pacificus* form one of two distinct mouth morphs which are also associated with specific feeding behaviors [16]. This irreversible developmental process results in the formation of either the stenostomatous (St) mouth type which is strictly microbivorous and is characterized by a narrower mouth opening and a single small dorsal tooth. Alternatively, animals can develop the eurystomatous (Eu) morph which promotes an omnivorous diet including predatory behaviors and is defined by a wider, shallower mouth structure, an enlarged dorsal tooth and an additional sub-ventral tooth [48] (Fig 3A). The majority of *P*. *pacificus* strains exhibit an Eu bias under most conditions assessed which suggests a propensity for these strains to aggressively compete with other nematodes in their environment via predatory interactions [22,49]. Importantly, the *P*. *pacificus* mouth morph fate is influenced by a panoply of well characterized genetic and environmental factors providing a mechanism to manipulate the mouth form and subsequently the *P*. *pacificus* predatory behavior [49–53]. The nuclear hormone receptor *Ppa-nhr-40* is required for the Eu mouth morph fate [51], and as such mutations in this gene result in 100% of animals forming the St mouth form. As all our selected strains are highly Eu, we mutated *Ppa-nhr-40* in both RSB001 and RSA075 which succeeded in abolishing their predatory behavior (Figs 3B, 3C, S3A and S3B).

With the *Ppa-nhr-40* ^RSB001^ and *Ppa-nhr-40* ^RSA075^ non-predatory variants established, we next explored the influence of their predatory behaviors on group selectivity and aggregate formation. Mixed *Ppa-nhr-40* ^RSB001^ and *Ppa-nhr-40* ^RSA075^ mutant cultures show higher frequencies of between strain aggregates than RSB001 and RSA075 wild type strains (Fig 3D–3F). However, to investigate this further, we also conducted assays between *nhr-40* mutants together with their rival strain Eu morph. With only one strain capable of predatory behaviors in these assays we predicted the Eu strain would dominate and form aggregates at the expense of its *Ppa-nhr-40* mutant opponent. Indeed, in both pairwise assays involving the St variant with its Eu rival, aggregation in the St strain was significantly impaired (Fig 3E–3F). Thus, phenotypic plasticity and its associated predatory interactions are key components influencing aggregation behaviors.

### Mutations in *self-1* are insufficient to disrupt aggregation

**Fig 4:**
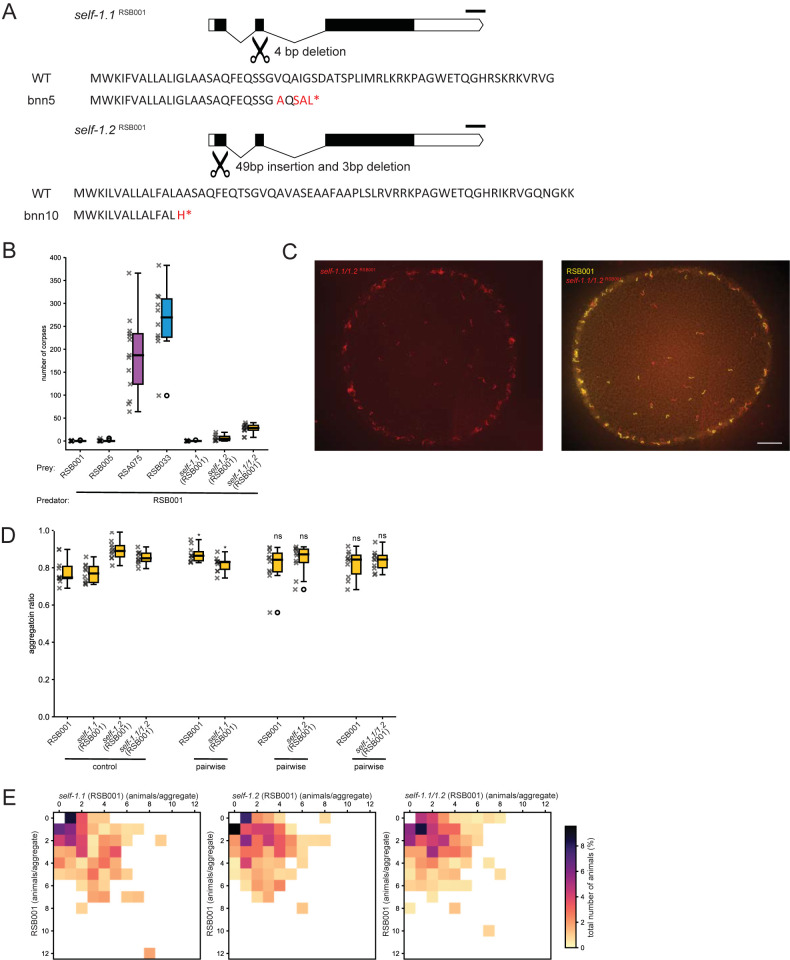
The kin-recognition component SELF-1 is dispensable for aggregate formation. (A) Predicted gene structure of *self-1*.*1* and self-1.2 in strain RSB001. CRISPR/Cas9 target sites are highlighted across both genes. Mutations generated resulted in severely truncated proteins and putative null mutants. Predicted wildtype protein sequences as well as associated mutations are shown. Scale bar = 100 bp, (Scissors image from openclipart.org). (B) Quantification of predation assays revealing the modest kin-killing phenotype caused by mutations in *self-1*. n = 21 for RSB001 prey, n = 13 for RSB005 prey, n = 12 for RSA075 prey and n = 10 for RSB033 prey, *self-1*.*1* ^RSB001^ prey, *self-1*.*2* ^RSB001^ prey and *self-1*.*1; self-1*.*2* ^RSB001^ prey. (C) Representative images of the aggregation phenotypes of the *self-1*.*1; self-1*.*2* ^RSB001^ mutant as well as mixed cultures of *self-1*.*1; self-1*.*2*^RSB001^ with RSB001. Scale bar = 2000 μm. (D) Quantification of aggregation ratios of *P*. *pacificus* cultures including of RSB001 and *self-1* mutants alone as well as mixed cultures of RSB001 together with *self-1* mutants. Mutations in *self-1*.*1; self-1*.*2*^RSB001^ are not sufficient to disrupt aggregation. Statistical tests were performed against control (Mann-Whitney-U-test with Bonferroni corrections). n = 10 per condition. (E) Heatmaps of pairwise interactions between RSB001 and *self-1*.*1* ^RSB001^, RSB001 and *self-1*.*2* ^RSB001^, and RSB001 and *self-1*.*1; self-1*.*2* ^RSB001^ revealing the proportion of each strain in an aggregate. Mixed RSB001 and the *self-1* mutants cultures aggregate together.

With aggregate formation observed between animals of the same genotype as well as between kin but not more distantly related strains, we next explored the importance of the *P*. *pacificus* molecular kin-recognition components for these interactions. The only molecular component involved in this process that has been described so far is *self-1* which encodes for a small peptide containing a hypervariable C-terminus that is thought to be important for generating between strain specificity. Mutations in *self-1* cause a mild kin-recognition defect resulting in predators erroneously killing their own offspring [21]. *self-1* has been previously identified in all of our four selected assay strains [22] (S4 Fig). As RSB001 has previously formed the basis of numerous molecular studies we focused on this strain [25,27,45]. We generated a putative *self-1* null mutant (*self-1*.*1*) in RSB001, however surprisingly no kin-recognition defect was observed (Fig 4A and 4B). Therefore, we analyzed the RSB001 genome further and identified a further potential *self-1* paralogue which shares 72.9% sequence identity at the amino acid level outside of the hypervariable domain (Fig 4A). This additional copy was designated *self-1*.*2* and a subsequent CRISPR/Cas9 induced putative null mutation successfully phenocopied the modest kin-defective phenotype previously described in *self-1* mutants in other strains (Fig 4A and 4B) [21]. Additionally, a *self-1*.*1; self-1*.*2* double mutant revealed a stronger kin-killing defect than the *self-1*.*2* single mutant alone (Fig 4B). Therefore, we next assessed if the kin-recognition defect associated with these *self-1* mutations was sufficient to also disrupt aggregation behaviors. In aggregation assays consisting of either mutants alone, or assays containing an equal amount of RSB001 together with *self-1* mutants, aggregation was maintained at wild type control levels despite the kin-recognition defect (Fig 4B–4E). Therefore, the mild kin-recognition defect caused by these mutations is insufficient to disrupt aggregation. With recent studies demonstrating that additional as yet unidentified components must also contribute to the kin-recognition signal [22], we predict that mutations in these other elements may result in stronger kin-recognition defects which could be sufficient to also influence and disrupt aggregation behavior.

### *P*. *pacificus* territorial biting prevents *C*. *elegans* aggregation

**Fig 5:**
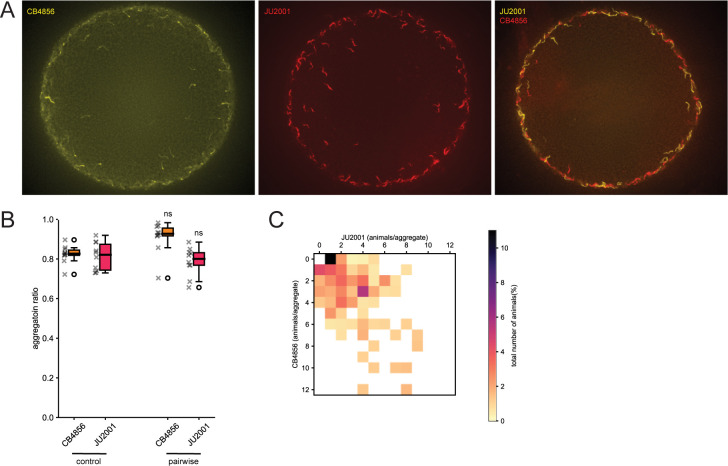
*C*. *elegans* pairwise interactions between aggregating strains. (A) Representative images of the aggregation phenotypes of the *C*. *elegans* strains CB4856 (Hawaiian) and JU2001 (La Reunion) controls as well as mixed cultures of CB4856 and JU2001 together. Scale bar = 2000 μm. (B) Quantification of aggregation ratios of *C*. *elegans* cultures including of CB4856 and JU2001 alone as well as mixed cultures of CB4856 and JU2001 together. Both *C. elegans* strains aggregate together abundantly. Statistical tests were performed against control (Mann-Whitney-U-test with Bonferroni corrections). n = 10 per condition. (C) Heatmap of pairwise interactions between of CB4856 and JU2001 revealing the proportion of each strain in an aggregate. Mixed CB4856 and JU2001 cultures form frequent aggregates together.

**Fig 6:**
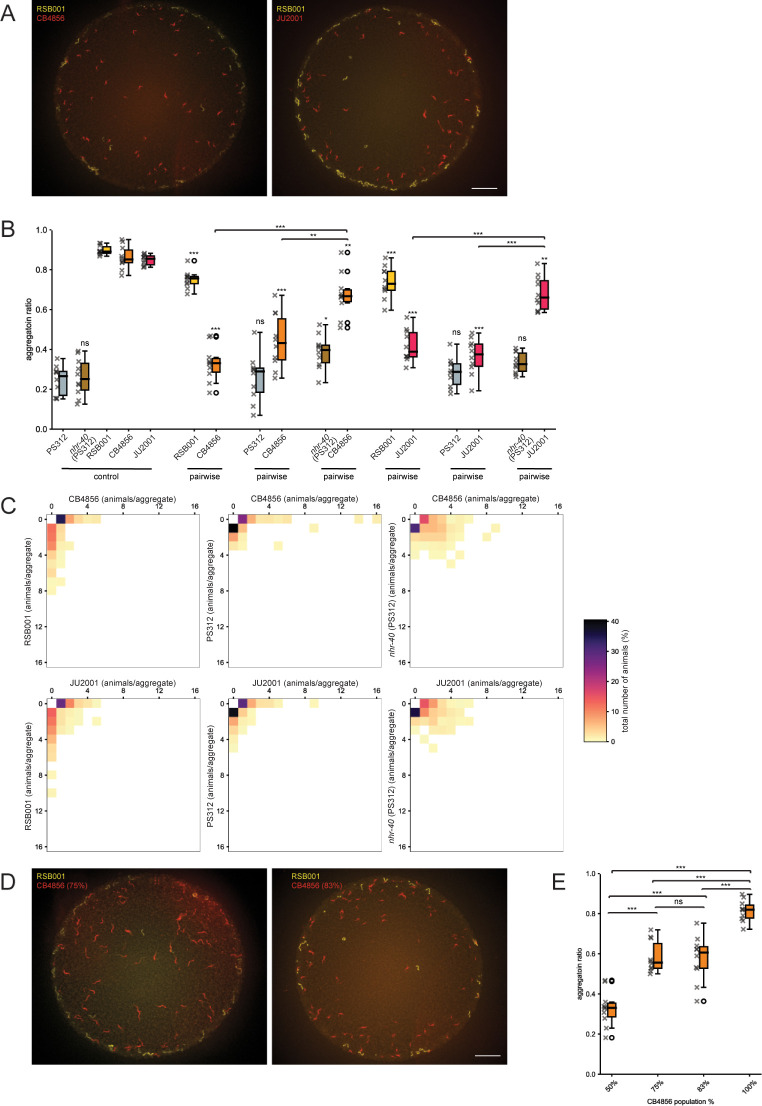
*pacificus* interferes with *C*. *elegans* aggregation behaviors. ***P*.** (A) Representative images of the aggregation phenotypes with *P*. *pacificus* in mixed cultures together with *C*. *elegans* strains CB4856 (Hawaiian) or JU2001 (La Reunion). Scale bar = 2000 μm. (B) Quantification of aggregation ratios of *P*. *pacificus* aggregating strain RSB001, solitary strain PS312, the St mutant strain *Ppa-nhr-40*^*PS312*^ and *C*. *elegans* CB4856 and JU2001 controls as well as mixed cultures of each *P*. *pacificus* strain together with each of the *C*. *elegans* strain. Both RSB001 and PS312 disrupts the aggregation ability of both *C*. *elegans* strains while *Ppa-nhr-40*^*PS312*^ does not. Statistical tests were performed against control, unless there are bars above the boxplots for any between-pair comparisons (Mann-Whitney-U-test with Bonferroni corrections). n = 10 per condition. (C) Heatmap of pairwise interactions between *P*. *pacificus* RSB001, PS312, and *Ppa-nhr-40*^*PS312*^ together with *C*. *elegans* CB4856 or JU2001 revealing the proportion of each strain in an aggregate. Increased aggregations were observed between the St mutant strain and *C*. *elegans* strains. (D) Representative images of the aggregation phenotypes with a reduced ratio of *P*. *pacificus* to *C*. *elegans* CB4856 in mixed cultures assays. Scale bar = 2000 μm. (E) Quantification of aggregation assays with a reduced ratio of *P*. *pacificus* RSB001 to *C*. *elegans* CB4856. Even a minimal *P*. *pacificus* presence is capable of shaping the aggregation behaviors of *C*. *elegans*. Statistical tests were performed against control, unless there are bars above the boxplots for between-pair comparisons (Mann-Whitney-U-test with Bonferroni corrections). n = 13 for 100% CB4856 and n = 10 for each of the other conditions.

Interactions between con-specific *P*. *pacificus* likely occur frequently in their natural habitat as strains compete to occupy their preferred ecological niche. Additionally, *P*. *pacificus* also comes into contact with other nematode species which frequently includes rhabditid species and rarely *C*. *elegans* although, as yet, *C*. *elegans* has not been found in the high-altitude environments where the clade B strains are located [54,55]. The *P*. *pacificus* predatory ability however has previously been implicated in territorial behaviors against *C*. *elegans* [19,56]. Therefore, we next assessed conspecific aggregation interactions between diverse *C*. *elegans* strains as well as the impact of *P*. *pacificus* on the aggregation ability of these wild *C*. *elegans* isolates. We selected two strains of *C*. *elegans* which both aggregate strongly, CB4856 isolated from the Hawaiian Islands, and JU2001 from La Reunion Island (Fig 5A). Utilizing the same fluorescent dye method employed to stain *P*. *pacificus* [47], we firstly explored pairwise aggregation assays between these con-specific *C*. *elegans* strains. Here, both strains successfully aggregated with one another at similar levels to with their own strain indicating the presence of another distantly related rival did not disrupt aggregation in *C*. *elegans* (Fig 5A–6C). Next, we challenged the *C*. *elegans* aggregating strains by pairing them with the aggregating *P*. *pacificus* strain RSB001, a non-aggregating *P*. *pacificus* strain PS312, or a *P*. *pacificus* St mutant strain *Ppa-nhr-40*^PS312^ which could therefore not predate. In these mixed species assays, we observed *C*. *elegans* failed to aggregate and instead behaved in a more solitary manner only when it was challenged with *P*. *pacificus* strains capable of predation (Fig 6A–6C). Finally, as *P*. *pacificus* strongly displaces *C*. *elegans* from aggregates, we analyzed the potency of *P*. *pacificus* for influencing these *C*. *elegans* behaviors by reducing the representation of *P*. *pacificus* present in mixed species pairwise assays. Here, we observed *C*. *elegans* aggregation behaviors were disrupted when only 17% of the assay population was *P*. *pacificus* (Fig 6D–6E). Additionally, under these conditions *C*. *elegans* aggregates were mostly only maintained when they consisted of large numbers of animals. Therefore, *C*. *elegans* aggregates above a density threshold may be sufficient to resist predation induced displacement and may represent an ecological relevant strategy to counter the effects of *P*. *pacificus* predation (S5A and S5B Fig). Thus, a minimal *P*. *pacificus* presence is capable of shaping the aggregation behaviors of other species such as *C*. *elegans*. These interactions may therefore represent a territorial behavior conferring an ecologically relevant and advantageous state for *P*. *pacificus* within their shared environmental niche.

## Discussion

Group behavior in nematodes including aggregate formation emerge from local interactions between individuals [33]. In *P*. *pacificus* however, an additional layer of complexity is evident through its predatory capacity which functions to broaden potential food opportunities and as a mechanism to remove competitors from their local environment [18–22] as well as its kin-recognition ability which prevents attacks on close relatives [21,22]. In our work, we show these elements combine to also impact their collective behaviors as aggregation is favored between kin while predatory attacks between distantly related con-specifics result in the displacement of their rivals and the induction of less preferable behavioral strategies. Moreover, in pairwise assays between distantly related strains, we also observed the frequency of monotypic aggregation increases. Importantly, the exclusion of potential competitors from optimal kin groupings is not unique to *P*. *pacificus* and has been observed in many organisms. This includes in slime molds which aggregate together in fruiting bodies under stressful conditions. This behavior depends on the between strains compatibility of the cell surface *csA* gene which ultimately facilitates admission to the fruiting body [5,57]. Additionally, in social yeast species, robust cell adhesion molecules promote efficient homogenous aggregate and biofilm formation while weak adhesive forces between non-kin result in their exclusion [2,58]. However, these mechanisms of selectivity and omission depend on preventing aggregations between non-kin which differs from our observations in *P*. *pacificus* whereby predation provides an active mechanism to displace competitors.

Subsequently, we also demonstrated that the aggressive displacement of potential competitors from aggregates is at least partially dependent on the predatory Eu mouth form. This is the most prevalent morph found in wild *P*. *pacificus* isolates including those from the high altitude adapted clade utilized in these studies [22,49]. As all strains of *P*. *pacificus* are capable of also developing the non-predatory St morph, aggregation behaviors are therefore theoretically possible even between highly divergent strains which may prove to be beneficial under certain environmental conditions. In addition to the role of *P*. *pacificus* predation, previous studies have identified several genetic components that are necessary for *P*. *pacificus* aggregation. In particular, mutations in the IFT system cause a solitary strain of *P*. *pacificus* to aggregate [25–27,37]. Therefore, in the future, it will be important to assess the impact of these mutations on con-specific aggregation behaviors which may aid in establishing more of the sensory mechanisms involved however, due to their substantial influence on mouth morph fate, these experiments will not be straightforward.

While predation and kin-recognition appear to play an integral role in aggregation behaviors in *P*. *pacificus*, neither behavior has been observed in *C*. *elegans*. Accordingly, we observed aggregation between distantly related social strains of *C*. *elegans* with no apparent effect on their behaviors. This is consistent with previous studies in which a mixed population of *C*. *elegans* including an aggregating strain and an evolutionary distinct solitary strain, each maintained their specific behavioral phenotypes when grown together [35]. However, in mixed assays between aggregating *C*. *elegans* and *P*. *pacificus* strains, aggregation was hindered in *C*. *elegans* while maintained in *P*. *pacificus*. Furthermore, the presence of relatively few *P*. *pacificus* predators proved sufficient to modulate the aggregation behavior in *C*. *elegans* suggesting a minimal amount of *P*. *pacificus* animals are capable of altering the group dynamics of a much larger competing nematode population. Additionally, as *P*. *pacificus* develops slower and have a smaller brood size than *C*. *elegans* [59,60], the ability to alter the behavior and outcompete other nematodes such as *C*. *elegans* may be highly beneficial. Moreover, this may represent an additional facete to the previously characterized *P*. *pacificus* territorial behaviors which have been shown to disrupt *C*. *elegans* ability to approach and enter shared bacterial lawns [19]. In the future, it will be essential to explore other effects which may influence these aggregation dynamics and which are likely to be influenced by a suite of sensory inputs. These may include signals as diverse as oxygen [25], kin-relevant signals [21], chemical messages such as ascarosides [61,62], and alarm pheromones which are released from injured nematodes and which may be triggered by the predatory events [63].

With our work revealing a previously unknown influence for kin-recognition and its associated predation on collective behaviors in *P*. *pacificus*, questions remain regarding what other behaviors may also be affected by these abilities. For example, recent work in *C*. *elegans* has demonstrated that their swimming gait is influenced by the presence of other nearby animals [64]. Additionally, more diverse collective behaviors including swarming have been described in *C*. *elegans* [32,33] and groups of both *C*. *elegans* and *P*. *pacificus* are capable of forming large 3D tower like structures thought to aid in their dispersal to new environments [54,65]. Therefore, how predation and kin-recognition may influence these other collective behaviors remains unknown and will form the basis of future studies. Thus, kin-recognition abilities in *P*. *pacificus* facilitate a diverse behavioral repertoire of interactions. This includes the territorial removal of competitors via predatory events, as well as between kin-aggregation in which individuals nepotistically favor grouping with relatives as rivals take on less preferable behavioral strategies. Furthermore, with the wealth of molecular tools available, *P*. *pacificus* offers a powerful means for understanding the genetic and neural mechanisms behind these kin-recognition mediated interactions and their evolution.

## Materials and methods

### Nematode Husbandry

All nematodes used were maintained on standard NGM plates on a diet of *Escherichia coli* OP50. All strains used in this study can be found in the S1 Table.

### Aggregation assays

Aggregation assays were utilized to quantify aggregation behaviors between different strains. Assay plates were prepared two days prior to the experiment. 6 cm NGM plates were seeded with 70 μl of OP50 bacteria and incubated at room temperature. All animals were maintained on NGM plates seeded with OP50 bacteria until freshly starved, resulting in an abundance of young larvae. These plates were washed with M9 and passed through two 20 μm filters to isolate pure cultures of larvae. They were centrifuged and transferred onto new NGM plates seeded with OP50 bacteria and incubated at 20°C for three days until they become young adults. They were then washed with M9 and transferred into 1.5 ml tubes, containing 50 μM of dye (CellTracker Green BODIPY Dye or CellTracker Orange CMRA Dye), and incubated on a rotator in dark at 20°C for 3 h. The worms were then washed with M9 four times and transferred into an unseeded NGM plate. For controls, 120 worms from one strain were picked and transferred to an assay plate, outside of the OP50 bacterial lawn. For pairwise aggregation assays, 60 worms per strain from two strains were transferred to an assay plate. The plates were incubated at 20°C in dark for 3 h, then imaged using a fluorescent microscope ZEISS Axio Zoom.V16 with ZEN (blue edition) software. Images were analyzed manually with Fiji. It was considered an aggregate when two or more animals were in contact along 50% of their body length. The staining with CellTracker dyes method was adapted from Werner et al [47] and pairwise aggregation assay was adapted from Moreno et al [25]. The number of animals in solitary state as well as in each aggregate are manually scored from a static image of the assay plate. The aggregation ratio is calculated by: (total number of animals of strain A in aggregates) / (total number of animals of strain A found on the assay plate).

### Off food aggregation assay

Off food aggregation assays were utilized to investigate aggregation behaviors of nematodes in an environment without bacterial food (OP50). Animals are well-fed and are washed prior to the experiment. 360 worms of the same strain were transferred on a 3.5 cm NGM plate. The plates were incubated at 20°C for 1 h. Images were taken using brightfield on microscope ZEISS Axio Zoom.V16 with ZEN (blue edition) software.

### CRISPR/Cas9 induced mutations

Mutations were induced in candidate genes via CRISPR/Cas9. Gene specific crRNA and universal trans-activating CRISPR RNA (tracrRNA) was purchased from Integrated DNA Technologies and 5 μL of each 100 μM stock mixed and denatured at 95°C for 5 min before cooling at room temperature to anneal. Cas9 (Integrated DNA Technologies) was added to the hybridized product and incubated at room temperature for 5 mins. This was subsequently diluted with TE buffer to a final concentration of 18.1 μM for the sgRNA and 2.5 μM Cas9. This was injected into the germline of the required *P*. *pacificus* strain. Eggs from injected P0s were recovered up to 16 h post injection. After hatching and 2 days’ growth these F1 were segregated onto individual plates until they had also developed sufficiently and egg laying had been initiated. The genotype of the F1 animals were subsequently analyzed via Sanger sequencing and mutations identified before re-isolation in homozygosis. sgRNAs and associated primers utilized in this study can be found in S2 Table.

### Mouth-form

Mouth form phenotyping was achieved by observation of the nematode buccal cavity using ZEISS Axio Zoom.V16 microscope with morph identities based on previous described species characteristics [49]. Final mouth-form frequencies are the mean of 3 independent replicates, each assaying 100 animals.

### Predation and Kin-recognition assays

Corpse assays facilitated rapid quantification of predatory behaviors between different strains. Prey were maintained on NGM plates seeded with OP50 bacteria until freshly starved, resulting in an abundance of young larvae. These plates were washed with M9 and passed through two 20 μm filters to isolate pure cultures of larvae. They were subsequently centrifuged before being deposited on to an unseeded assay plate by pipetting 1.5 μl of *P*. *pacificus* or 1.0 μl of *C*. *elegans* larval pellet on to a 6 cm NGM unseeded plate. Five predatory nematodes were screened for the appropriate mouth morph and added to assay plates for prey assays. They were then permitted to feed on the prey for 2 h and the plate was subsequently screened for the presence of corpses. As *self-1* mutants are only mildly kin-recognition defective it is necessary to conduct predation assays over 20 h before screening the plate for the presence of corpses.

### Statistical analysis

Statistical tests were performed using Python with Scipy and statsmodels libraries. Box plot represents the first quartile (Q1) to the third quartile (Q3) of the data with a line at the median (Q2). Q1, Q2, and Q3 are the values which lie at 25%, 50% and 75% of the data points. Whiskers represent the range in which most values are found, whereas values outside this range are presented as outliers. The statistical tests were always performed against the control condition of the same strain. Non-significant (ns), p-value ≤ 0.05 (*), p-value ≤ 0.01 (**), p-value ≤ 0.001 (***), p-value ≤ 0.0001 (****).

## Supporting information

S1 Fig The solitary *C*. *elegans* strain N2 and social CB4856 both do no aggregate in the absence of bacteria. The *P*. *pacificus* PS312 solitary strain also does not aggregate in the absence of food however the social RSB001 continues to aggregate. Scale bar = 2000 μm.
(PDF)

S2 Fig (A) Vital dyes allow fluorescent labelling of distinct populations for identification in mixed cultures. Scale bar = 200 μm. (B) Aggregation is not affected by either CellTracker Green BODIPY Dye or CellTracker Orange CMRA Dye staining. Aggregation for RSB001 is shown. (C) All strains form frequent aggregates under standard laboratory conditions and are not affected by CellTracker Orange CMRA Dye (left image) or CellTracker Green BODIPY Dye (right image). Scale bar = 2000 μm. (D) Heatmaps of aggregation size distribution in control aggregation assays of each strain. (E) Quantification of aggregation ratios in the non-aggregating strain PS312 and the aggregating strain RSB001. Minimal transient aggregates occur in PS312. n = 10 per strain.
(PDF)

S3 Fig (A) Gene structure and CRISPR target site for *Ppa-nhr-40* mutations in both RSB001 and RSA075. Mutations were successfully generated in both strains. Scale bar = 1kb. (Scissors image from openclipart.org) (B) Mutations result in a frame shift in both RSB001 and RSA075 strains which leads to a putative premature stop codon and a truncated protein.
(PDF)

S4 Fig *self-1* is present in all four strains used in the study. This includes variable copy numbers and distinct hypervariable regions in RSB001, RSB005, RSA075 and RSB033. (PDF)

S5 Fig (A) Representative aggregation assay with a reduced ratio of *P*. *pacificus* RSB001 to *C*. *elegans* CB4856. Occasionally *C*. *elegans* aggregates were established consisting of large numbers of *C*. *elegans* (marked *) which may be sufficient to resist predation induced displacement. Scale bar = 2000 μm. (B) Heatmaps of pairwise interactions between reduced ratio of *P*. *pacificus* RSB001 and increased ratio of *C*. *elegans* CB4856 revealing the proportion of each strain in an aggregate.
(PDF)

S1 Table List of all strains used and alleles associated with the mutations. (XLSX)

S2 Table List of primers and CRISPR/Cas9 associated sequences for generating mutants. (XLSX)

S1 Movie Movie showing pairwise assay between two competing strains. RSA075 is stained yellow and RSB001 is stained in red. (AVI)

S2 Movie Movie showing biting interaction between adults of the distantly related strains RSB001 and RSA075. A RSB001 predator (top most animal) approaches and makes contact with the RSA075 aggregate. Nose contact and a probable bite causes one RSA075 to temporarily leave the aggregate before returning.
(MOV)
