# Supplementary figures and images for: Kin-recognition and predation shape collective behaviors in the cannibalistic nematode Pristionchus pacificus
Source: PLoS Genet. 2023 Dec 14;19(12):e1011056. doi: 10.1371/journal.pgen.1011056 (PMC10721034; doi:10.1371/journal.pgen.1011056)

S1 Fig.

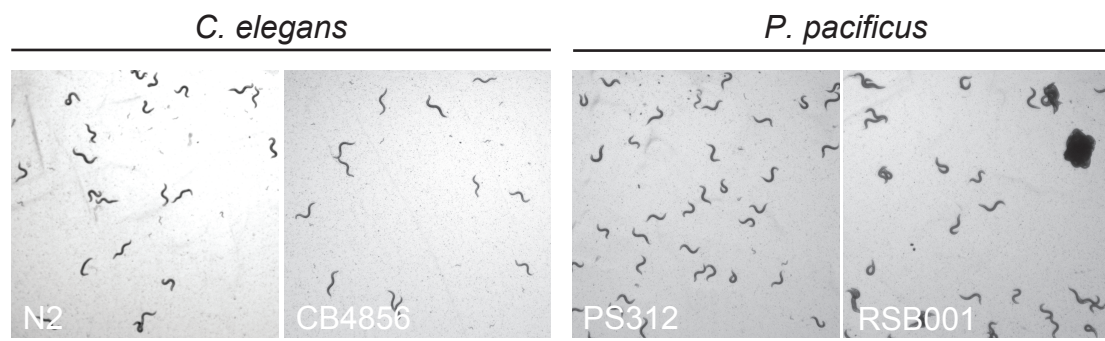

Supplement: S1 Fig — The P. pacificus PS312 solitary strain also does not aggregate in the absence of food however the social RSB001 continues to aggregate. Scale bar = 2000 μm. (PDF) [file pgen.1011056.s001.pdf]

S2 Fig.

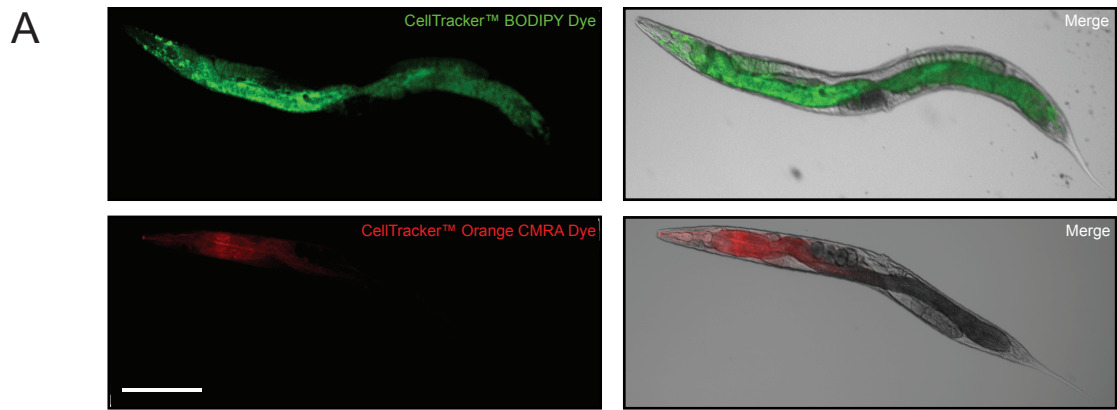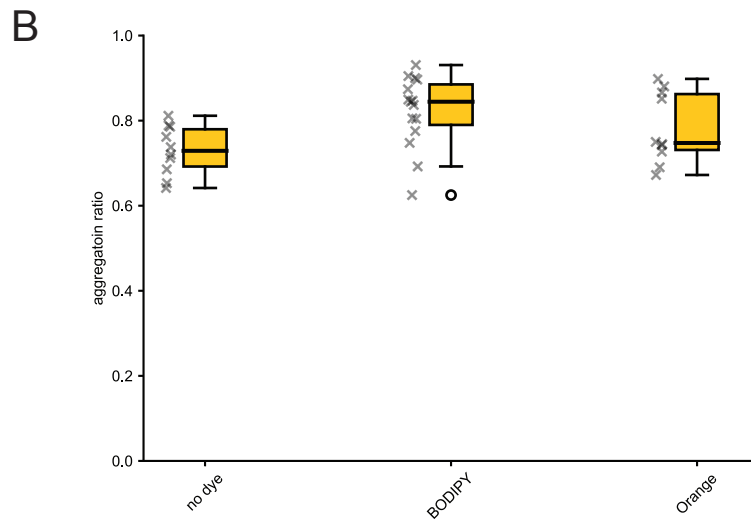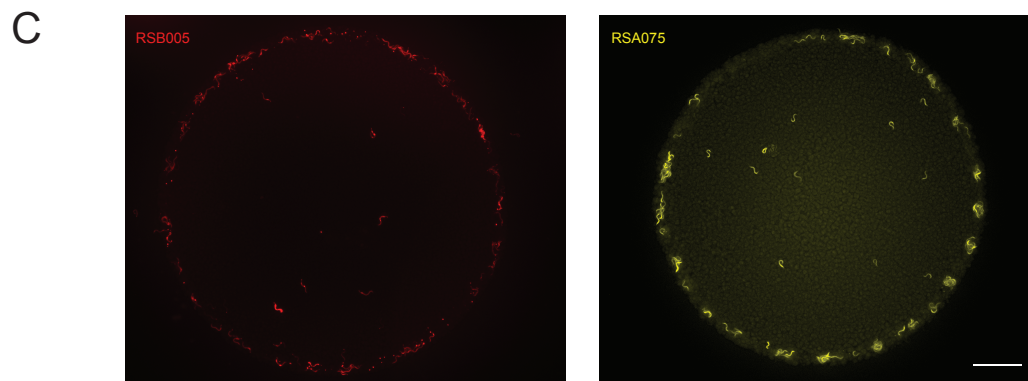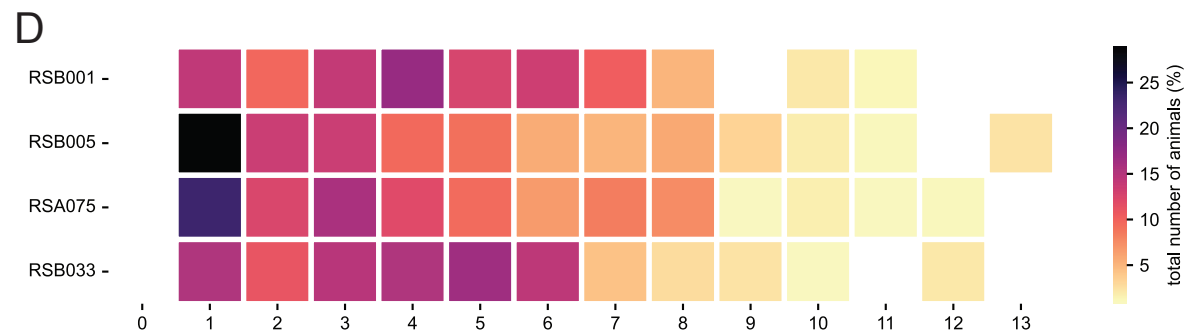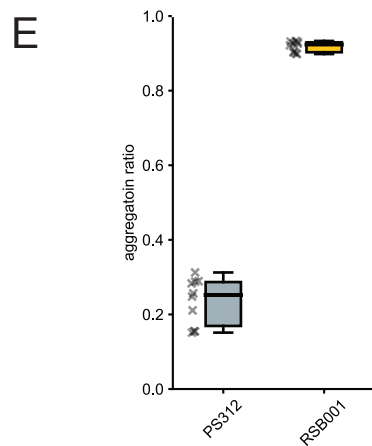

Supplement: S2 Fig — (A) Vital dyes allow fluorescent labelling of distinct populations for identification in mixed cultures. Scale bar = 200 μm. (B) Aggregation is not affected by either CellTracker Green BODIPY Dye or CellTracker Orange CMRA Dye staining. Aggregation for RSB001 is shown. (C) All strains form frequent aggregates under standard laboratory conditions and are not affected by CellTracker Orange CMRA Dye (left image) or CellTracker Green BODIPY Dye (right image). Scale bar = 2000 μm. (D) Heatmaps of aggregation size distribution in control aggregation assays of each strain. (E) Quantification of aggregation ratios in the non-aggregating strain PS312 and the aggregating strain RSB001. Minimal transient aggregates occur in PS312. n = 10 per strain. (PDF) [file pgen.1011056.s002.pdf]

S5 Fig.

A

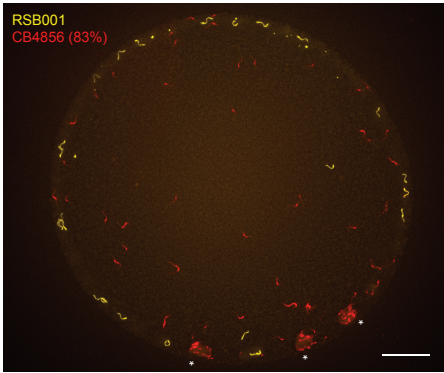

B

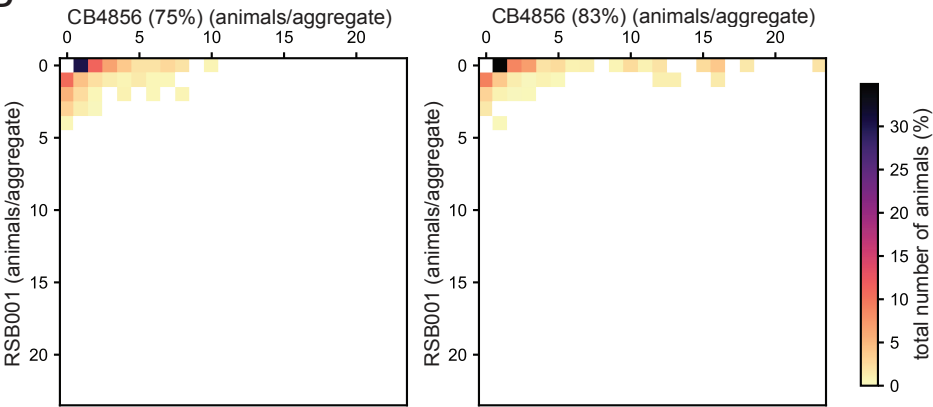

Supplement: S5 Fig — (A) Representative aggregation assay with a reduced ratio of P. pacificus RSB001 to C. elegans CB4856. Occasionally C. elegans aggregates were established consisting of large numbers of C. elegans (marked *) which may be sufficient to resist predation induced displacement. Scale bar = 2000 μm. (B) Heatmaps of pairwise interactions between reduced ratio of P. pacificus RSB001 and increased ratio of C. elegans CB4856 revealing the proportion of each strain in an aggregate. (PDF) [file pgen.1011056.s005.pdf]
