# Supplementary material for: Kin-recognition and predation shape collective behaviors in the cannibalistic nematode Pristionchus pacificus
Source: PLoS Genet. 2023 Dec 14;19(12):e1011056. doi: 10.1371/journal.pgen.1011056 (PMC10721034; doi:10.1371/journal.pgen.1011056)
Supplement: S3 Fig — (A) Gene structure and CRISPR target site for Ppa-nhr-40 mutations in both RSB001 and RSA075. Mutations were successfully generated in both strains. Scale bar = 1kb. (Scissors image from openclipart.org) (B) Mutations result in a frame shift in both RSB001 and RSA075 strains which leads to a putative premature stop codon and a truncated protein. (PDF) [file pgen.1011056.s003.pdf]

S3 Fig.

A

Ppa-nhr-40

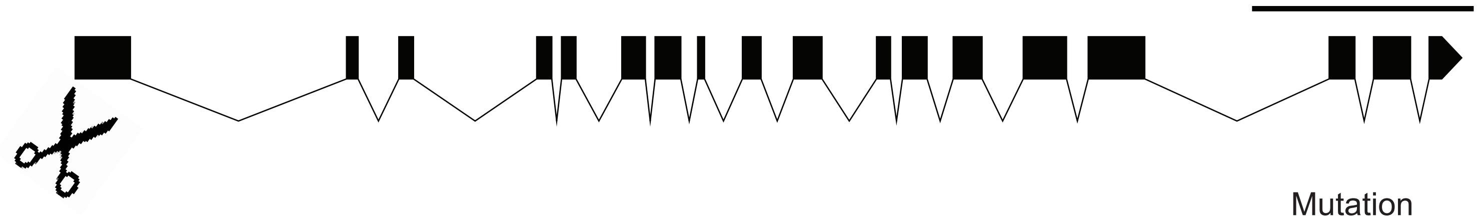

		Mutation
Ppa-nhr-40	GAAGATTCCGGAGGG-AACCCTCTGTGTCGT	Wild type
Ppa-nhr-40 RSB001 *(bnn38)*	GAAGATTCCGGAGGGGAACCCTCTGTGTCGT	1bp insertion
Ppa-nhr-40 RSA075 *(bnn40)*	GAAGATTCCGGAG-----CCCTCTGTGTCGT	4bp deletion

B

Ppa-nhr-40

MEIYGRCDYTTTHRVEIYTRREKIPEGTLCVVCDDSASGIHYSVASCNGCKTFFRRALVN
KQTFTCQFEGKCLVGKNVRCVCRSCRLKKCFEQGMDPKAIQHDRDKIRYTKVLKREKEAL
KAKKEAERESMMMKVKEEIGSPGGLECCDIPSTSKGNPFSFLSPMEMILSRFLSNEPSND
LDSNLRELMRIEKKVVEVRNAYRYDDQISTIWNMYIGSRAMLSEDDWLSATTQQKPMLFS
LSERQEPAQVSEPPKTSPYRCARITPWSLREWFQRDLTLMMEWVKLIPGINDLITSDKVI
LQKNFALTFAVYQLTFYTMDTTVLSGDDFAPSELSSLEERLKSLKRRSDSRSTPPPPAPC
LSSTINNELMNLANSLYKQPLAKRIKDEILEDEPTCSAYLRKLTEQASLLATSSPSTSSF
VNSPIDSAFLMKNGLTAVSSFSEGINQFPSNMPPLPPTMSTGLPGALSNCLASSLQAASK
NMTSLPTTLPTFPSGFFSALHNSPASLGFPPSPLLASLTASPLITSSPLMAQSPLMAQSP
LMASLTMPSHSLPSTFPNFLPPPVPAHLALPPPSLPTLLIPLLPQPRPLPVNQLPPAPVP
IREKTPDIDVETVSMDGRSEGRGSRAIDTTDSRDEVLFRVPASLPRSISVTSSSDVIKPF
NLMSMRSPDDPSRTVKMDSILEKMSVSPAEQMTIPSTIVSSVMSTSTSSNNIKDEPESPE
EMTSSNSSSVIKNTGDVTEITKEPKVHKPQAENRTMFEDEETPCDPTPEAQIANRINYPD
GTFIERDKERPFNDELYGLLIDGIWKIFRRENVDQETFVLFKMMSFFNTELTHKGDHKLS
EDGVKYVEKMRQKMYTQLLLHLQKTKGKDGLRIFSSLLLMGSTIARVRNALRKMFTMTSI
FVPSNDLVDQLILRDNDERVPSPSAFSIYHPVY*

Ppa-nhr-40 RSB001 *(bnn38)*

MEIYGRCDYTTTHRVEIYTSGQNSDKTLILMLFPGREKIPEGNPLCRL*

Ppa-nhr-40 RSA075 *(bnn40)*

MEIYGRCDYTTTHRVEIYTSGQNSDKTLILMLFPGREKIPEPSVSSVMIRPLAFTILSLRAM
DAKPSSEELS*
