# Supplementary material for: Kin-recognition and predation shape collective behaviors in the cannibalistic nematode Pristionchus pacificus
Source: PLoS Genet. 2023 Dec 14;19(12):e1011056. doi: 10.1371/journal.pgen.1011056 (PMC10721034; doi:10.1371/journal.pgen.1011056)
Supplement: S4 Fig — (PDF) [file pgen.1011056.s004.pdf]

S4 Fig.

		hypervariable domain
SELF-1^{RSB001}	MWKILVALLALIGLAASAQFEQSSGVQAIGSDATSPLIMRLKRKPAGWETQGHRSKR	KVRVG----
SELF-1^{RSB005}	MWKILVALLALIGLAASAQFEQSSGVQAIGSDATSPLIMRLKRKPAGWETQGHRSKR	KVRVG----
SELF-1.1^{RSA075}	MWKILVALLALIGLAASAQFEQSSGVQAIGSDATSPLIMRLKRKPAGWETQGHRSKR	V--------
SELF-1.2^{RSA075}	MWKILVALLALIGLAASAQFEQSSGVQAIGSDATSPLIMRLKRKPAGWETQGHRSKR	I--------
SELF-1^{RSB033}	MWKILVALLALIGLAASAQFEQSSGVQAIGSDATSPLIMRLKRKPAGWETQGHRSKR	IPVGRGHGK
